# A meta-analysis of ethnic differences in pathways to care at the first episode of psychosis

**DOI:** 10.1111/acps.12254

**Published:** 2014-02-28

**Authors:** K K Anderson, N Flora, S Archie, C Morgan, K McKenzie

**Affiliations:** 1 Social and Epidemiological Research, Centre for Addiction and Mental Health (CAMH)Toronto, ON, Canada; 2 Department of Psychiatry and Behavioural Neurosciences, McMaster UniversityHamilton, ON, Canada; 3 Section of Social Psychiatry, Institute of Psychiatry, King's College LondonLondon, UK; 4 Department of Psychiatry, University of TorontoToronto, ON, Canada

**Keywords:** schizophrenia and disorders with psychotic features, health services accessibility, population groups

## Abstract

**Objective:**

We sought to systematically review the literature on ethnic differences in the likelihood of general practitioner (GP) involvement, police involvement, and involuntary admission on the pathway to care of patients with first-episode psychosis (FEP).

**Method:**

We searched electronic databases and conducted forward and backward tracking to identify relevant studies. We calculated pooled odds ratios (OR) to examine the variation between aggregated ethnic groups in the indicators of the pathway to care.

**Results:**

We identified seven studies from Canada and England that looked at ethnic differences in GP involvement (*n *= 7), police involvement (*n *= 7), or involuntary admission (*n *= 5). Aggregated ethnic groups were most often compared. The pooled ORs suggest that Black patients have a decreased likelihood of GP involvement (OR = 0.70, 0.57–0.86) and an increased likelihood of police involvement (OR = 2.11, 1.67–2.66), relative to White patients. The pooled ORs were not statistically significant for patients with Asian backgrounds (GP involvement OR = 1.23, 0.87–1.75; police involvement OR = 0.86, 0.57–1.30). There is also evidence to suggest that there may be ethnic differences in the likelihood of involuntary admission; however, effect modification by several sociodemographic factors precluded a pooling of these data.

**Conclusion:**

Ethnic differences in pathways to care are present at the first episode of psychosis.

Anderson KK, Flora N, Archie S, Morgan C, McKenzie K. A meta-analysis of ethnic differences in pathways to care at the first episode of psychosis.

**Summations**

- Ethnic differences in pathways to care are well documented for psychiatric disorders generally, but research on first-episode psychosis (FEP) specifically has been inconsistent. We found evidence to suggest that differences in pathways to care between aggregated ethnic groups are present at the first episode.
- Most prior studies have focused on differences in pathways to care for aggregated ethnic groups, with little consideration of place of origin or immigration status.
- Many prior studies were underpowered and were not designed specifically to look at ethnic differences in pathways to care, and most did not find significant differences between groups. However, once the data from these studies are meta-analyzed, the findings suggest that Black patients are less likely to have GP involvement and more likely to have contact with police on the pathway to care, as compared with White patients.

**Considerations**

- Pathways to care are highly dependent on the social, cultural, and health service context, which needs to be considered when interpreting the results of the meta-analysis.
- More comprehensive studies that are designed and powered to examine differences in pathways to care are needed to elucidate the relative contributions of place of origin, culture, and immigration status.

## Introduction

Early detection of first-episode psychosis (FEP) and reductions in treatment delay are important service developments in psychiatry. Understanding the routes that people take to obtain care may facilitate the development of services that decrease the time from first symptoms to effective treatment. The pathway to care that a person takes often involves a complex series of contacts with service providers in an effort to obtain help for the symptoms of psychosis. Social, cultural, and health services factors are important in shaping both the direction and the duration of the care pathway 1.

Ethnicity has the potential to influence the nature and direction of the pathway to care. Ethnicity describes the social group a person belongs to based on factors such as language, religion, and place of origin 2. It will have an impact on illness models, social connections, and consequently care pathways. An individual's ethnic background influences decisions about whether and how to seek help, as well as the array of services and supports that are available to the patient throughout the help-seeking process 3,4. Perceived differences between ethnic groups may also impact interactions with service providers 5. Other factors known to covary with ethnic group, such as poverty and discrimination, may also influence the help-seeking process 6, and economic and language barriers may impede access to care 7.

Ethnic differences in pathways to care are well documented for chronic psychiatric disorders, with African and Caribbean origin groups typically experiencing more complex and coercive pathways 8,9. However, it is important to consider the pathways to care at the first episode specifically, as initial experiences and interactions with health services could have a lasting impact on subsequent help-seeking attempts, engagement with services, and adherence with treatment. Additionally, an extended period of treatment delay at the first episode is a potentially modifiable risk factor for poor clinical and functional outcomes 10–12 that may represent disparities that vary along ethnic lines. A prior systematic review of the literature looked at several sociodemographic determinants of the pathway to care in FEP 13, including ethnicity, but we are not aware of any reviews that have examined each of the determinants in depth or meta-analysed the findings from prior studies.

### Aims of the study

The objective of this study was to systematically review the literature on ethnic differences in pathways to care among patients with first-episode psychosis to determine whether the differences observed in psychiatric disorders generally are also present at the first episode of psychosis specifically. Because the pathways to care are affected by the availability and accessibility of services within a given health system, and there are differences in service provision between low-, middle-, and high-income countries, we restricted this review to studies conducted in high-income countries to increase comparability across the studies.

## Material and methods

### Choice of indicators

Prior studies have examined a wide array of indicators of the pathway to care, and these differ substantially in how they are defined and operationalized 13. Therefore, we chose to focus our analysis on three factors that have been shown to have important implications for help-seeking behaviour, treatment delay, and long-term trajectories:

- General practitioner (GP) involvement has been previously shown to reduce the likelihood of contact with police and emergency services, but has also been associated with a longer treatment delay 14–16;
- Police and criminal justice involvement on the pathway to care has been hypothesized to be associated with an increased likelihood of service disengagement 17;
- Involuntary admission has been shown to be associated with dissatisfaction with services for patients and their family members 18, as well as symptoms of PTSD in patients 19 and distress among family members 20. This could lead to an avoidance of health services for subsequent mental health concerns 21.

We hypothesize that the ethnic differences in pathways to care that are observed in psychiatric disorders generally 8,9 will also be present at the first episode, such that minority groups will be less engaged with primary care services and have an increased likelihood of police involvement and involuntary admissions.

### Systematic review

Our search strategy was developed in consultation with a librarian and involved an electronic search of the MEDLINE (1950–2012), HealthStar (1966–2012), EMBASE (1980–2012) and PsycINFO (1967–2012) databases using the OvidSP platform with search terms specific to each database (Appendix 1). We also searched the Web of Knowledge using key words.

We obtained further studies by manually searching personal files and the reference lists of all included papers. We also conducted forward citation searching using Web of Knowledge to identify papers that had cited the included studies. When abstracts or unpublished papers were retrieved in our search, we contacted the corresponding authors to determine whether the work had been subsequently published. We regularly updated all segments of the literature search, with the final update in September 2012.

Each study was examined by one reviewer for the following inclusion criteria: i) the study measures the pathways to care of people with FEP; ii) the article examines the pathways to care by ethnic group; iii) the study was conducted in a high-income country 22; and iv) the findings were published in a peer-reviewed journal. Where inclusion of a particular paper was unclear, the paper was brought to the other investigators for discussion and consensus. We did not impose any restrictions with respect to date, study design, or language of publication.

For all papers that met the inclusion criteria, two independent reviewers extracted data on key elements of study design, the definition and measurement of pathways to care, the method of assigning ethnicity, and frequency counts and proportions for key indicators. We assigned a quality assessment (QA) score to each paper using a rating scale adapted from the Newcastle-Ottawa Scale 23 and a tool used in previously published systematic reviews on ethnic differences in pathways to care 8,24 (Table1). Discrepancies between the reviewers were resolved by consensus. Where important methodological details were missing from the paper, we consulted prior studies from the research group to obtain the missing information.

**Table 1:** Rating system for methodological quality adapted from the Newcastle-Ottawa Scale 23 and from previously used scales from systematic reviews of ethnic differences in pathways to care 8,24

Rating criteria		
1. Representativeness of participants	−	Selected group/No description of the derivation of the sample
	•	Somewhat representative (ex. clinical sample)
	+	Truly representative (ex. complete catchment area sample)
2. Non-participation rate	−	High rate and no description of differences/Non-participation not described
	•	High rate and differences described
	+	Low rate and differences described
3. Adequacy of sample size	−	No power calculation or inadequate sample to detect ethnic differences
	+	Authors demonstrate that the sample was adequately powered to detect ethnic differences
4. Definition of first-episode psychosis	−	Not described
	•	Based on first hospitalization
	+	Based on duration of antipsychotic treatment or first presentation to a clinical setting
5. Ascertainment of ethnicity	−	Not reported
	•	Third-party report (e.g. Staff categorization, name-based method)
	+	Self-reported ethnicity
6. Classification of ethnicity	−	Ethnic groups dichotomized (e.g. White vs. others)
	•	Use of aggregated groups (ex. African origin and Caribbean origin combined as ‘Black’)
	+	All analyses carried out on specific ethnic groups without aggregation
7. Adjustment for confounding factors	−	None
	•	Age and/or gender only
	+	Other comorbidities or risk factors for the outcome of interest*
8. Definition of pathways to care	−	Definition of pathways to care unclear (ex. no description of start/endpoint, type of contacts)
	+	Clear definition of pathways to care
9. Ascertainment of pathways to care	−	Not described/Chart review or third-party report only
	•	Patient report only
	+	Patient report corroborated with chart review or third-party information
10. Measurement of pathways to care	−	Not described/Non-systematic method used for measuring pathways to care
	+	Use of a standardized measurement tool for measuring pathways to care
11. Same method of ascertainment for entire sample	−	No
	+	Yes

−, Criteria not met; •, Criteria partially met; +, Criteria satisfied.

* Risk factors include sociodemographic and clinical variables; Comorbidities include drug and alcohol use and other psychiatric conditions.

### Meta-analysis

We conducted meta-analyses to quantify the between-group variation in the likelihood of GP involvement and police involvement on the pathway to care. We were unable to meta-analyse the data on involuntary admissions, as there was evidence in several studies of effect modification by age 25, gender 25,26, and socioeconomic status 27. Thus, we decided that it would be inappropriate to compute a single common effect estimate that ignores the impact of these factors, and we present a qualitative summary of the findings across the different studies. Of note, effect modification has also been tested for in both GP involvement and police involvement, and the investigators did not find evidence of an interaction with other variables 28.

Only two studies examined separate ethnic groupings. In these studies, the data were presented separately for Black-African (people who identified as Black and were born in Africa), Black-Caribbean (people who identified as Black and were born in the Caribbean) and Black-British (people who identified as Black and were born in the UK) patients, as well as for White-British (people who identified as White and were born in the UK) and White-Other (people who identified as White and were born outside of the UK) patients 25,28,29. In most studies, aggregated ethnic groupings were presented in which all those who were from ‘Black’ or ‘White’ subgroups were analysed together. We summed the frequency counts for these estimates and calculated odds ratios for Black and White groups, respectively, to allow comparability with the other studies that examined aggregated ethnic groupings. We also meta-analysed the data for the ‘Asian’ group, including the study by Cole and colleagues that lumped the ‘Asian’ group with ‘Other’ 30. We did not meta-analyse the data for the ‘Other’ ethnic groupings due to the limited availability of data and the inherent heterogeneity in such a classification.

We calculated summary odds ratios using the *metan* procedure in Stata/IC 11.0 (StataCorp LP, College Station, TX, USA). Statistical heterogeneity was assessed using the *I*^2^ statistic, with values of 25%, 50%, and 75% suggestive of low, moderate, and high heterogeneity respectively 31. There was a high likelihood of methodological and contextual heterogeneity due to the different definitions used for pathways to care and for ethnicity, as well as the different health service contexts of each of the studies; therefore, we opted to use a random effects model to compute the summary effect estimates 32.

To examine the influence of each individual study on the overall effect estimate, we conducted a sensitivity analysis that calculated the pooled OR and CI after omitting each study in turn using the *metaninf* procedure. Sensitivity analyses were used to examine the influence of the country of origin of the study, as well the influence of specific items from the quality assessment that were most often missed across the studies (*n *≥ 3 studies not meeting the criterion). Finally, publication bias was explored by generating funnel plots using the *metafunnel* procedure.

## Results

**Figure 1:**
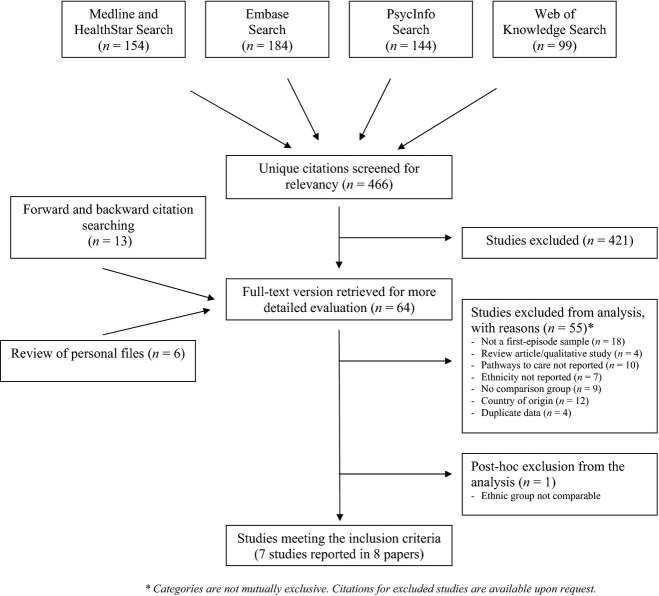
Flow chart of the systematic review search strategy and exclusion process.

We identified 64 potential articles that were reviewed for inclusion, and we excluded 55 that did not meet the inclusion criteria (reasons listed in Fig.1). One additional study was excluded *post hoc* because the ethnic group classification was not comparable to the other studies, as the authors compared the care pathways of an Aboriginal group (Maori) with the non-Aboriginal population 33.

In total, eight papers presenting data from seven different studies compared the pathways to care of ethnic minority groups to the majority population [the findings of Morgan and colleagues were reported in two articles 25,28]. Data were available from all studies for the meta-analysis of GP involvement (pooled sample: White = 1004; Black = 682; Asian = 175) and police involvement (pooled sample: White = 1019; Black = 684; Asian = 180), and five studies presented findings on the likelihood of involuntary admission.

### Study characteristics

The characteristics of the included studies are summarized in Tables2 and 3, and the quality assessment ratings for study methodology are presented in Table4. The studies used cross-sectional designs and were conducted in Canada or England. The size of the samples varied substantially, ranging from 93 to 775 participants (median across studies = 199). The two Canadian studies defined the first episode of psychosis based on duration of medication use, and the five studies from England defined it based on first contact with services (Table2).

**Table 2:** Characteristics of studies included in the review (*n *= 7)

Study	*n*	Source of sample	Source of data	Diagnostic criteria (tool)	% Non-affective	Definition of first-episode psychosis
Canada						
Anderson et al. 14	309 (with ethnicity data)	Catchment area based early intervention programme	Medical Records Patient interview Family interview Clinician interview	DSM-IV (SCID)	72	Psychotic symptoms in a patient who had received less than one consecutive month of prior antipsychotic treatment
Archie et al. 34	199 (with ethnicity data)	Early intervention services across four sites	Medical records Patient interview Family interview Clinician interview	DSM-IV (SCID)	100	Psychotic symptoms in a patient who had received less than 1 month of prior antipsychotic treatment
England						
Burnett et al. 27*	100	Catchment areas for two health districts	Medical records Patient interview Family interview	CATEGO (PSE)	100	First contact with health services or criminal justice agencies for schizophrenia
Cole et al. 30	93	Psychiatric catchment area of a hospital	Patient interview Family interview	ICD-9 (PSE)†	67†	First contact with psychiatric services for a psychotic disorder
Ghali et al. 29	775	Early intervention services across eight sites	Patient interview Medical records Collateral history	Not Described	Not Described	First contact with psychiatric services for affective or non-affective psychosis
Harrison et al. 26	131	Catchment area of psychiatric services	Medical records Patient interview Family interview	ICD-9 (PSE)	89	First contact with psychiatric services for psychosis
Morgan et al. 25	462	Cases from secondary or tertiary services in catchment area	Medical records Patient interview Family interview	ICD-10 (SCAN)	74	Patients presenting to services for the first time with an ICD-10 diagnosis of psychosis‡

DSM, Diagnostic and Statistical Menu of Mental Disorders; SCID, Structured Clinical Interview for DSM; ICD, International Classification of Diseases; SCAN, Schedules for Clinical Assessment in Neuropsychiatry.

* Not included in meta-analysis of involuntary status.

† As per prior paper 47.

‡ As per subsequent paper 48.

**Table 3:** Measurement of pathways to care and ethnicity for all studies included in the review (*n *= 7)

Study	Start point for pathway to care	Endpoint for pathway to care	Instrument	Ethnicity measurement	Ethnicity categories (*n*)	GP involvement, % (*n*)	Police involvement, % (*n*)	Involuntary admission, % (*n*)
Canada								
Anderson et al. 14	Prodrome to psychosis	Entry into an early intervention programme	CORS	Staff assigned based on place of origin	White (196)	34 (57)*	46 (90)	–
					Black (42)	29 (10)*	57 (24)	–
					Asian (40)	44 (15)*	40 (16)	–
					Other (31)	27 (7)*	39 (12)	–
Archie et al. 34	After the onset of psychosis	Entry into an early intervention service	CORS	Self-report	White (121)	30 (35)*	13 (14)*	72 (54)*
					Black (31)	37 (10)*	23 (5)*	69 (9)*
					Asian (25)	25 (6)*	20 (4)*	38 (6)
					Other (22)	33 (7)*	10 (2)*	86 (12)*
England								
Burnett et al. 27	Contact which resulted in admission to hospital or psychiatric services		PPHS	Self-report	White (37)	51 (19)	22 (8)	N/A
					Afro-Caribbean (37)	38 (14)	35 (13)	N/A
					Asian (24)	54 (13)	4 (1)	N/A
Cole et al. 30	Not described	First contact with psychiatric services	Ad-hoc for study purposes	Self-report Staff assigned †	White (39)	69 (27)	38 (15)	28 (11)
					Black (38)	68 (26)	45 (17)	39 (15)
					Asian and Other (16)	81 (13)	44 (7)	19 (3)
Ghali et al. 29	After the onset of psychosis	Entry into an early intervention programme	Electronic Audit Tool (MiData)	Staff assigned	White-British (215)	54 (99)*	17 (31)*	–
					Other White (123)	39 (40)*	23 (23)*	–
					Black-British (169)	42 (63)*	27 (41)*	–
					Black-Caribbean (28)	26 (7)*	33 (9)*	–
					Black-African (150)	44 (60)*	36 (48)*	–
					South Asian (90)	53 (41)*	15 (12)*	–
Harrison et al. 26	Not described	First contact with psychiatric services	PPHS	Staff assigned ‡	Afro-Caribbean (42)	60 (25)	19 (8)	45 (19)
					General Population (89)	75 (67)	7 (6)	21 (19)
Morgan et al. 25	Not described	First contact with psychiatric services	PPHS	Self-report Staff assigned Case notes	White-British (237)	52 (122)*	19 (44)*	27 (64)
					Other White (33)	52 (17)	21 (7)	30 (10)
					Black-Caribbean (128)	40 (51)*	36 (46)*	52 (66)
					Black-African (64)	34 (22)	41 (26)	55 (35)

GP, general practitioner; CORS, Circumstances of Onset and Relapse Schedule; PPHS, Psychiatric and Personal History Schedule; N/A, Data not available from the author; –, Indicator not studied.

* Does not sum to total sample *n* due to missing data.

† As per prior paper 47.

‡ As per prior paper 49.

**Table 4:** Quality assessment ratings for studies included in the systematic review (*n *= 7)

	Anderson et al. 14	Archie et al. 34	Burnett et al. 27	Cole et al. 30	Ghali et al. 29	Harrison et al. 26	Morgan et al. 25
1. Representativeness of participants	•	•	+	+	•	+	+
2. Non-participation rate	+	•	−	+	•	•	+
3. Adequacy of sample size	−	−	−	−	−	−	−
4. Definition of first-episode psychosis	+	+	+	+	+	+	−
5. Ascertainment of ethnicity	•	+	+	+	•	•	+
6. Classification of ethnicity	•	•	•	•	+	−	+
7. Adjustment for confounding factors	+	+	+	+	+	•	+
8. Definition of pathways to care	+	+	+	−	+	−	−
9. Ascertainment of pathways to care	+	+	+	+	+	+	+
10. Measurement tool for pathways to care	+	+	+	+	+	+	+
11. Same method of ascertainment for entire sample	−	+	+	+	+	−	−

−, criteria not met; •, criteria partially met; +, criteria satisfied.

All studies used a standardized instrument for measuring pathways to care, as well as multiple data sources to corroborate information. The endpoint for the pathway to care was either contact with psychiatric services (*n *= 4) or admission to an early intervention programme (*n *= 3). However, several of the studies (*n *= 3) did not explicitly report the starting point of the pathway to care (Table3).

Four studies used a self-report measure of ethnicity, and three used staff assignment. Two studies performed analyses on specific ethnic groups without aggregation 25,28,29, and one study considered the immigration status of participants by distinguishing between first- and second-generation people of African, Caribbean and European origin 29. The classifications of ethnicity that were used are shown in Table3.

None of the included studies met all of our QA criteria (Table4). The most common problems across the studies were as follows: non-representative sample (*n *= 3); non-participation rate high or not described (*n *= 4); not using a self-report measure for ethnicity, or not describing how it was measured (*n *= 3); aggregation of ethnic groups (*n *= 5); not providing a clear description of the pathway to care (*n *= 3); and not using the same method of ascertainment for the entire sample (*n *= 3). The effects of these factors on the overall conclusions were explored in the sensitivity analyses of the quality assessment items (described below). None of the studies demonstrated that the sample size was adequate for detecting ethnic differences in pathways to care (Table4).

### Ethnic differences in pathways to care

#### General practitioner involvement

**Figure 2:**
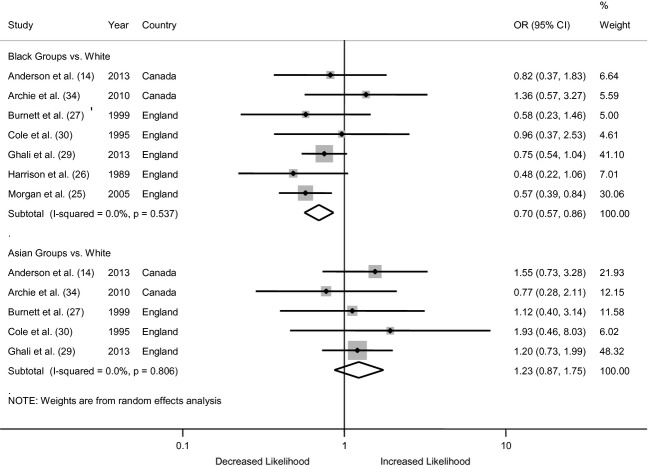
Forest plot from the meta-analysis of seven studies showing the individual and pooled odds ratios for differences in the likelihood of general practitioner (GP) involvement on the pathway to care. The area of the shaded box on the individual studies indicates the relative weight of each in the meta-analysis.

All seven of the studies included in our review used some indicator of GP involvement. We calculated the odds of GP involvement using the proportions taken from the seven studies, and these are presented in Fig.2. The pooled odds ratio across all the studies indicates that Black patients were significantly less likely to have GP involvement on their pathway to care, relative to White patients (OR = 0.70, 0.57–0.86). There was no evidence of differences in the likelihood of GP involvement for Asian groups (OR = 1.23, 0.87–1.75). The *I*^2^ estimates suggest no statistical heterogeneity in the data for either group (*I*^2^ = 0%).

For both analyses, the conclusions remain unchanged in the sensitivity analysis (data not shown), which recalculates the summary effect estimate after removing each individual study, in turn. In the sensitivity analyses by country, the conclusions remain unchanged for both Black and Asian groups in the studies from England (Black OR = 0.66, 0.53–0.82; Asian OR = 1.24, 0.81–1.91), but there are no significant differences between groups in the findings from Canada (Black OR = 1.03, 0.57–1.87; Asian OR = 1.19, 0.61–2.31). The finding of a reduced likelihood of GP involvement is no longer statistically significant when we reanalyse the data using only the studies that used a self-report measure for ethnicity (QA Criterion #5 OR = 0.72, 0.48–1.06), that had a clearly defined definition of pathways to care (QA Criterion #8 OR = 0.79, 0.60–1.03), or that used the same method of ascertainment for the entire sample (QA Criterion #11 OR = 0.80, 0.60–1.05). Omitting studies that did not meet the remaining QA criteria that were commonly missed (Criteria #1, 2, 6) did not change the conclusions of the meta-analysis (data not shown). Finally, the asymmetrical nature of the funnel plot for GP involvement indicates the possibility of publication bias for data from Asian groups (data not shown).

#### Contact with police and the criminal justice system

**Figure 3:**
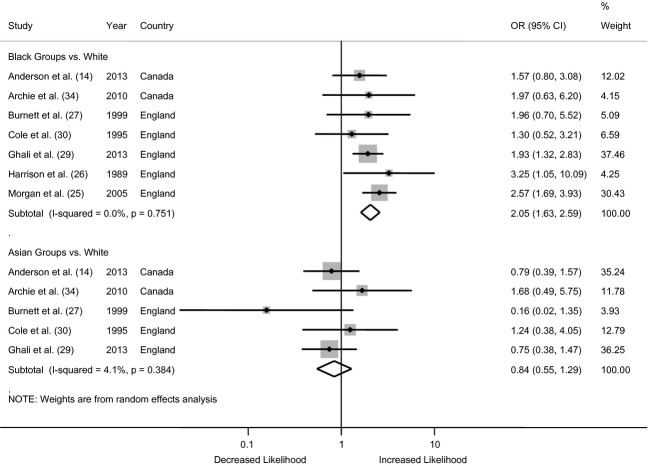
Forest plot from the meta-analysis of seven studies showing the individual and pooled odds ratios for differences in the likelihood of police involvement on the pathway to care. The area of the shaded box on the individual studies indicates the relative weight of each in the meta-analysis.

All of the studies included in our review used some indicator of police and criminal justice involvement. We calculated the odds of police and criminal justice involvement using the proportions taken from the seven studies, and these are presented in Fig.3. For the comparison of Black and White groups, the pooled odds ratio across all the studies indicates that Black patients were twice as likely to have police involvement in their pathway to care, relative to White patients (OR = 2.05, 1.63–2.59). There was no evidence of an excess risk of police involvement for Asian groups (OR = 0.84, 0.55–1.29). The *I*^2^ estimates suggest no heterogeneity in the data from Black patients (*I*^2^ = 0%) and very little heterogeneity in the data for Asian groups (*I*^2^ = 4.1%).

For both analyses, the conclusions remain unchanged in the sensitivity analysis (data not shown), which recalculates the summary effect estimate after removing each individual study, in turn. In the sensitivity analyses by country, the conclusions remain unchanged for both Black and Asian groups in the studies from England (Black OR = 2.14, 1.66–2.76; Asian OR = 0.73, 0.34–1.57), but are not significant for the findings from Canada (Black OR = 1.67, 0.93–2.97; Asian OR = 0.96, 0.50–1.86), likely due to the limited sample size. Omitting studies that did not meet the QA criteria that were most frequently not satisfied (Criteria #1, 2, 5, 6, 8, 11) did not change the conclusions of the meta-analysis. Finally, the asymmetrical nature of the funnel plot for police involvement indicates the possibility of publication bias for both groups (data not shown).

#### Involuntary admission

Five of the included studies looked at ethnic differences in the likelihood of involuntary admissions. Archie and colleagues found that patients of Asian ethnicity in Canada were less likely to have an involuntary admission, relative to other groups 34. Among the studies from England, both Harrison and colleagues and Morgan and colleagues found that Black-Caribbean patients were more likely to be admitted involuntarily 25,26, and Morgan additionally found that Black-African patients were also more likely to have an involuntary admission, relative to White patients 25. Both studies found evidence of effect modification by gender, although in opposite directions. Specifically, Harrison and colleagues found that Black-Caribbean females, but not males, were more likely to have an involuntary admission 26, whereas Morgan and colleagues report a significant association for males only 25. The latter study also found evidence of effect modification by age, with younger Black-Caribbean patients having a much higher odds of involuntary admission than older Black-Caribbean patients, relative to the White group 25. Burnett and colleagues did not find significant evidence of ethnic differences in involuntary admissions overall, but did find evidence of effect modification when the interaction between ethnicity and other sociodemographic factors were considered. Specifically, they found that the risk of involuntary admission was higher for White males with low education, for Black males who were living alone and for Asian patients who were living in public housing 27. Cole and colleagues from England found that Black patients were more likely to have an involuntary admission, but this finding did not reach statistical significance 30.

## Discussion

Our systematic literature review and meta-analysis on ethnic differences in the pathway to care in FEP found significant differences in the likelihood of both GP involvement and police involvement for Black patients in Canada and England, relative to White patients. Black patients were less likely to have GP involvement and more likely to have police involvement on their pathway to care. We did not find evidence of differences for patients of Asian backgrounds; however, we had a very limited availability of data for these meta-analyses. We also found evidence to suggest that there may be ethnic differences in the likelihood of involuntary admission for Black patients with FEP; however, there was significant effect modification by sociodemographic factors, and we therefore opted not to meta-analyse these data. It is noteworthy that the included studies also reported ethnic differences in other indicators of the pathways to care, including the type of first contact 34, the source of referral into psychiatric services 28, the total number of contacts on the care pathway 14,34, family involvement in help-seeking 28 and the likelihood of contact with emergency services 29.

Prior reports of ethnic differences in pathways to care in FEP have been inconsistent 13. There has been speculation that ethnic disparities in service use may arise only after the first episode, owing to increased stigma from within minority communities or negative experiences with services received at the first episode 27,30. However, it is likely that these inconsistent reports are due to the inadequate sample size of many of the prior studies. The results of our meta-analysis suggest that when the data from these studies are pooled, there is a significantly reduced likelihood of GP involvement and an increased likelihood of police involvement for Black patients relative to White. Even with the pooling of data in the meta-analysis, the Asian groups are underrepresented and small numbers may explain the lack of significance in the pooled effect estimates. The fact that these findings are not being driven by any one study suggests that the prior studies reporting no significant difference were likely underpowered. Indeed, none of the included studies demonstrated that they had obtained a sufficient sample for detecting ethnic differences in pathways to care. Based on the proportions reported in each of the papers, only the studies by Morgan and colleagues 28 and Ghali and colleagues 29 are adequately powered to detect an odds ratio of at least two (as found in the meta-analysis of police involvement) for a Black vs. White comparison of groups. Despite their large sample sizes, these studies are still underpowered for examining differences in some of the specific ethnic groupings without aggregation.

The included studies typically assessed differences in pathways to care for aggregated ethnic groups, with little consideration of place of origin or immigration status. The ethnic groupings within countries tend to be culturally heterogeneous; therefore, it is important to also include factors such as place of origin and immigration status in the discourse on pathways to care. The studies by both Ghali and colleagues and Morgan and colleagues analysed differences in pathways to care with no aggregation of ethnic groups and did find evidence of differences in the likelihood of negative care pathways between patients of Caribbean origin and those of African origin 25,28,29. These studies have also reported differences in treatment delay between specific ethnic groups 29,35. A study from the Netherlands that did not meet inclusion criteria for our review has found that first- and second-generation immigrants with FEP tend to be referred to mental health services by emergency services more often than native-born individuals, and the risk is higher for second-generation immigrants relative to first-generation immigrants 16. Similarly, the study by Ghali and colleagues found differences between the White-immigrant group relative to the White-British group 29. However, in contrast to the study from the Netherlands, Ghali and colleagues found that first-generation African and Caribbean groups had a higher risk of contact with emergency services and the criminal justice system than their second-generation Black-British peers 29. Such differences across immigrant groups could arise due to language barriers, a lack of knowledge regarding local availability of mental health services, or an increased likelihood of help-seeking from alternative healers 36. The true mechanism underlying differences in pathways to care is likely to involve a complex interaction between population groupings, socio-economic and cultural influences, and immigration status; however, no studies to date have been designed or adequately powered to be able to disentangle the relative contributions of these facets.

Six of the seven studies included in our review adjusted effect estimates for potential confounding factors in the association between ethnicity and pathways to care (Table2). Although much of the literature on the determinants of pathways to care in FEP has been inconclusive, sociodemographic and clinical factors that have been previously found to be predictive of care pathways include gender, living alone at the time of onset, family involvement in help-seeking, mode of onset of psychosis, premorbid functioning, and psychopathology 13,37,38. It has been suggested that the observed ethnic differences in pathways to care may be better accounted for by indicators of social support and isolation 27, and two of the studies included in our review found evidence of effect modification by gender 25,26. There is evidence to suggest that there may be ethnic differences in sociodemographic factors among patients with FEP 39,40, as well as ethnic differences in clinical presentation 41,42. Consequently, a failure to account for the moderating or mediating effects of these variables through the use of multivariate models and interaction terms will distort the true association between ethnicity and pathways to care.

Our findings are limited by a number of factors. There is currently no validated tool for measuring pathways to care 43, and the included studies used a variety of different methods of defining and operationalizing the indices. We were also only able to identify seven studies that reported data on ethnicity and pathways to care, despite the large number of studies to date that have examined care pathways in FEP 13. The included studies are therefore not representative of the varying social, cultural and health service contexts across the totality of evidence on pathways to care. Furthermore, our findings are not generalizable in countries that do not structure their healthcare system around GPs. For example, in the United States, the GP is not used as a contact on the pathway to care 44. The concept of ethnicity remains challenging because of social, political, historical, and geographical influences that contextualize and, at times, change the boundaries and the meanings of group identity 45. Nevertheless, it is important to try and understand social inequalities along these lines as they may relate to how institutions, services, and society treat different groups.

The results of our systematic review and meta-analyses indicate that prior studies examining the association between ethnicity and pathways to care have been limited by underpowered samples, and that between-group differences in negative and coercive care pathways are present at the first episode. More detailed studies that are designed and powered to examine ethnic differences in pathways to care are needed to elucidate the relative contributions of immigration, culture, and social inequalities. Additionally, the discourse on the impact of ethnicity on pathways to care would benefit from a more detailed examination of the complex mechanisms behind this association. This could include: the use of pathway mapping 46, rather than dichotomizing data which results in a loss of information; qualitative approaches to further our understanding of the reasons behind ethnic differences in pathways to care; or multi-level approaches that additionally consider factors at the population level, such as stigma or local mental health legislation. This detailed documentation of the pathways to care of different ethnic groups is crucial for the design and implementation of culturally sensitive and equitable mental health services.

## Appendix 1

### Terms used for Medline search strategy

[exp. Schizophrenia and Disorders with Psychotic Features/ OR]

exp. Affective Disorders, Psychotic/ OR

psychosis.mp OR

psychotic disorder$.mp OR

schizophreni$.mp OR

severe mental illness$.mp]

AND

ethnic$.mp OR

visible minorit$.mp OR

ethno$.mp OR

immigra$.mp OR

migration.mp OR

afro$.mp OR

africa$.mp OR

caribbean.mp OR

black.mp OR

europ$.mp OR

white.mp]

AND

[exp. Referral and Consultation/ OR

exp. Health Services Accessibility/ OR

pathways to care.mp OR

pathways to health care.mp OR

pathways to mental health care.mp OR

pathways to psychiatric care.mp OR

pathways to services.mp OR

pathways to health services.mp OR

pathways to mental health services.mp OR

pathways to psychiatric services.mp]

exp, Item searched as a MeSH term with explosion; mp, Item searched as a keyword; $, Term may have any combination of endings.

## References

[b1] Rogler LH, Cortes DE (1993). Help-seeking pathways: a unifying concept in mental health care. Am J Psychiatry.

[b2] Porta M (2008). A dictionary of epidemiology.

[b3] Cauce AM, Domenech-Rodríguez M, Paradise M (2002). Cultural and contextual influences in mental health help seeking: a focus on ethnic minority youth. J Consult Clin Psychol.

[b4] Commander MJ, Cochrane R, Sashidharan SP, Akilu F, Wildsmith E (1999). Mental health care for Asian, black and white patients with non-affective psychoses: pathways to the psychiatric hospital, in-patient and after-care. Soc Psychiatry Psychiatr Epidemiol.

[b5] McGovern D, Hemmings P (1994). A follow-up of second generation Afro-Caribbeans and White British with a first admission diagnosis of schizophrenia: attitudes to mental illness and psychiatric services of patients and relatives. Soc Sci Med.

[b6] Jarvis GE (2007). The social causes of psychosis in North American psychiatry: a review of a disappearing literature. Can J Psychiatry.

[b7] Snowden LR, Yamada AM (2005). Cultural differences in access to care. Annu Rev Clin Psychol.

[b8] Bhui K, Stansfeld S, Hull S, Priebe S, Mole F, Feder G (2003). Ethnic variations in pathways to and use of specialist mental health services in the UK. Systematic review. Br J Psychiatry.

[b9] Van Os J, McKenzie K, Jones P (1997). Cultural differences in pathways to care, service use and treated outcomes. Curr Opin Psychiatry.

[b10] Marshall M, Lewis S, Lockwood A, Drake R, Jones P, Croudace T (2005). Association between duration of untreated psychosis and outcome in cohorts of first-episode patients: a systematic review. Arch Gen Psychiatry.

[b11] Norman RM, Malla AK (2001). Duration of untreated psychosis: a critical examination of the concept and its importance. Psychol Med.

[b12] Perkins DO, Gu H, Boteva K, Lieberman JA (2005). Relationship between duration of untreated psychosis and outcome in first-episode schizophrenia: a critical review and meta-analysis. Am J Psychiatry.

[b13] Anderson KK, Fuhrer R, Malla AK (2010). The pathways to mental health care of first-episode psychosis patients: a systematic review. Psychol Med.

[b14] Anderson KK, Fuhrer R, Schmitz N, Malla AK (2013a). Determinants of negative pathways to care and their impact on service disengagement in first-episode psychosis. Soc Psychiatry Psychiatr Epidemiol.

[b15] Anderson KK, Fuhrer R, Wynant W, Abrahamowicz M, Buckeridge DL, Malla A (2013b). Patterns of health services use prior to a first diagnosis of psychosis: the importance of primary care. Soc Psychiatry Psychiatr Epidemiol.

[b16] Boonstra N, Sterk B, Wunderink L, Sytema S, De Haan L, Wiersma D (2012). Association of treatment delay, migration and urbanicity in psychosis. Eur Psychiatry.

[b17] Compton MT (2005). Barriers to initial outpatient treatment engagement following first hospitalization for a first episode of nonaffective psychosis: a descriptive case series. J Psychiatr Pract.

[b18] Leavey G, King M, Cole E, Hoar A, Johnson-Sabine E (1997). First-onset psychotic illness: patients' and relatives' satisfaction with services. Br J Psychiatry.

[b19] Tarrier N, Khan S, Cater J, Picken A (2007). The subjective consequences of suffering a first episode psychosis: trauma and suicide behaviour. Soc Psychiatry Psychiatr Epidemiol.

[b20] Boydell J, Onwumere J, Dutta R (2014). Caregiving in first-episode psychosis: social characteristics associated with perceived “burden” and associations with compulsory treatment. Early Interv Psychiatry.

[b21] Swartz MS, Swanson JW, Hannon MJ (2003). Does fear of coercion keep people away from mental health treatment? Evidence from a survey of persons with schizophrenia and mental health professionals. Behav Sci Law.

[b22] The World Bank (2012). http://data.worldbank.org/about/country-classifications.

[b23] Wells G, Shea B, O'Connell D http://www.ohri.ca/programs/clinical_epidemiology/oxford.asp.

[b24] Sass B, Moffat J, Bhui K, McKenzie K (2009). Enhancing pathways to care for black and minority ethnic populations: a systematic review. Int Rev Psychiatry.

[b25] Morgan C, Mallett R, Hutchinson G (2005a). Pathways to care and ethnicity. 1: sample characteristics and compulsory admission. Report from the AESOP study. Br J Psychiatry.

[b26] Harrison G, Holton A, Neilson D, Owens D, Boot D, Cooper J (1989). Severe mental disorder in Afro-Caribbean patients: some social, demographic and service factors. Psychol Med.

[b27] Burnett R, Mallett R, Bhugra D, Hutchinson G, Der G, Leff J (1999). The first contact of patients with schizophrenia with psychiatric services: social factors and pathways to care in a multi-ethnic population. Psychol Med.

[b28] Morgan C, Mallett R, Hutchinson G (2005b). Pathways to care and ethnicity. 2: source of referral and help-seeking. Report from the AESOP study. Br J Psychiatry.

[b29] Ghali S, Fisher HL, Joyce J (2013). Ethnic variations in pathways into early intervention services for psychosis. Br J Psychiatry.

[b30] Cole E, Leavey G, King M, Johnson-Sabine E, Hoar A (1995). Pathways to care for patients with a first episode of psychosis. A comparison of ethnic groups. Br J Psychiatry.

[b31] Higgins JPT, Thompson SG, Deeks JJ, Altman DG (2003). Measuring inconsistency in meta-analyses. BMJ.

[b32] Field AP, Gillett R (2010). How to do a meta-analysis. Br J Math Stat Psychol.

[b33] Turner M, Smith-Hamel C, Mulder R (2006). Pathways to care in a New Zealand first-episode of psychosis cohort. Aust N Z J Psychiatry.

[b34] Archie S, Akhtar-Danesh N, Norman R, Malla A, Roy P, Zipursky RB (2010). Ethnic diversity and pathways to care for a first episode of psychosis in Ontario. Schizophr Bull.

[b35] Morgan C, Fearon P, Hutchinson G (2006a). Duration of untreated psychosis and ethnicity in the AESOP first-onset psychosis study. Psychol Med.

[b36] Hansson EK, Tuck A, Lurie S, McKenzie K, Task Group of the Services Systems Advisory Committee of the Mental Health Commission of Canada (2010). http://www.mentalhealthcommission.ca/SiteCollectionDocuments/Key_Documents/en/2010/Issues_Options_FINAL_English.

[b37] Chien VH, Compton MT (2008). The impact of mode of onset of psychosis on pathways to care in a hospitalized, predominantly African-American, first-episode sample. Early Interv Psychiatry.

[b38] Opjordsmoen S, Friis S, Melle I (2010). A 2-year follow-up of involuntary admission's influence upon adherence and outcome in first-episode psychosis. Acta Psychiatr Scand.

[b39] Morgan C, Abdul-Al R, Lappin JM (2006b). Clinical and social determinants of duration of untreated psychosis in the AESOP first-episode psychosis study. Br J Psychiatry.

[b40] Mallett R, Leff J, Bhugra D, Pang D, Zhao JH (2002). Social environment, ethnicity and schizophrenia. A case-control study. Soc Psychiatry Psychiatr Epidemiol.

[b41] Veling W, Selten J-P, Mackenbach JP, Hoek HW (2007). Symptoms at first contact for psychotic disorder: comparison between native Dutch and ethnic minorities. Schizophr Res.

[b42] Van der Ven E, Bourque F, Joober R, Selten J-P, Malla AK (2012). Comparing the clinical presentation of first-episode psychosis across different migrant and ethnic minority groups in Montreal, Quebec. Can J Psychiatry.

[b43] Singh SP, Grange T (2006). Measuring pathways to care in first-episode psychosis: a systematic review. Schizophr Res.

[b44] Compton MT, Esterberg ML, Druss BG, Walker EF, Kaslow NJ (2006). A descriptive study of pathways to care among hospitalized urban African American first-episode schizophrenia-spectrum patients. Soc Psychiatry Psychiatr Epidemiol.

[b45] Bonham VL, Warshauer-Baker E, Collins FS (2005). Race and ethnicity in the genome era: the complexity of the constructs. Am Psychol.

[b46] Gater R, de Almeida E, Sousa B (1991). The pathways to psychiatric care: a cross-cultural study. Psychol Med.

[b47] King M, Coker E, Leavey G, Hoare A, Johnson-Sabine E (1994). Incidence of psychotic illness in London: comparison of ethnic groups. BMJ.

[b48] Fearon P, Kirkbride JB, Morgan C (2006). Incidence of schizophrenia and other psychoses in ethnic minority groups: results from the MRC AESOP Study. Psychol Med.

[b49] Harrison G, Owens D, Holton A, Neilson D, Boot D (1988). A prospective study of severe mental disorder in Afro-Caribbean patients. Psychol Med.

